# A phase I/Ib study of OTSGC-A24 combined peptide vaccine in advanced gastric cancer

**DOI:** 10.1186/s12885-018-4234-8

**Published:** 2018-03-27

**Authors:** Raghav Sundar, Sun Young Rha, Hiroki Yamaue, Masahiro Katsuda, Koji Kono, Hyo Song Kim, Chan Kim, Kousaku Mimura, Ley-Fang Kua, Wei Peng Yong

**Affiliations:** 1 Department of Haematology-Oncology, National University Health System, 5 Lower Kent Ridge Road, Main Building Level 2, Singapore, S119074 Singapore; 2 Yonsei Cancer Center, Seoul, South Korea; 3 Wakayama Medical University Hospital, Wakayama, Japan; 4 Cancer Science Institute, National University of Singapore, Singapore, Singapore; 5 Department of Gastrointestinal Tract Surgery, Fukushima Medical University, Fukushima, Japan; 6 Department of Advanced Cancer Immunotherapy, Fukushima Medical University, Fukushima, Japan; 7 Department of Progressive DOHaD Research, Fukushima Medical University, Fukushima, Japan

**Keywords:** OTSGC-A24, Cancer vaccine, Phase I, Gastric cancer

## Abstract

**Background:**

We conducted a phase I/Ib, open-label, single-arm trial to assess the safety, tolerability and optimal scheduling regimen of OTSGC-A24 cancer vaccine in patients with advanced gastric cancer.

**Methods:**

Patients with advanced gastric cancer with HLA-A*24:02 haplotype were included in this study. OTSGC-A24 was administered at 1 mg in 3-weekly (3w), 2-weekly (2w), and weekly (1w) cohorts to evaluate the safety, immunological response and schedule. Based on the highest specific cytotoxic T lymphocyte (CTL) induction rate at 4 weeks, using the ELISPOT test, cohorts were expanded to define the optimal dosing schedule for OTSGC-A24.

**Results:**

In this study, 24 advanced gastric cancer patients with HLA-A*24:02 haplotype were enrolled and treated in 3 cohorts (3w cohort: 3; 2w cohort: 11 and 1w cohort: 10 patients). The most common adverse events were decreased appetite (29%), diarrhea (21%), myalgia (25%). The most common treatment-related adverse event was injection site erythema (25%). No dose-limiting toxicities were observed in any cohort and OTSGC-A24 was well tolerated. Positive CTL responses after vaccination were observed in 15 patients (75%) at 4 weeks: 3w cohort (33%), 2w cohort (88%), 1w cohort (78%). At 12 weeks, 18 patients had responded (90%); 3w cohort (100%), 2w cohort (100%), 1w cohort (78%). The best radiological was stable disease (40%). Median progression free survival was 1.7 months (95% CI: 1.4 to 3.5) and median overall survival was 5.7 months (95% CI 3.8 to 8.6).

**Conclusions:**

OTSGC-A24 combined peptide cancer vaccine was well tolerated. Significant responses in CTL were observed and the recommended phase 2 dose is 1 mg OTSGC-A24 sub-cutaneous, every 2 weeks. Although no radiological response was observed, a respectable overall survival was achieved, consistent with other immunotherapy agents being investigated in gastric cancer.

**Trial registration:**

ClinicalTrials.gov Identifier: NCT01227772, Date registered: 21 Oct 2010.

## Background

Gastric cancer (GC) is the second most common cancer in the world. The 5-year overall survival (OS) for patients with unresectable GC due to locally advanced disease or metastatic spread ranges from 5 to 15% [1, 2]. In this group of patients, palliative chemotherapy has been demonstrated to prolonged survival when compared with best supportive care [3]. Although taxanes and irinotecan are often used in the second-line setting, after progression on first-line platinum-based therapy, treatment toxicity precludes majority of patients from receiving chemotherapy due to poor PS and organ function. Hence, there is a need to develop a less toxic alternative in these groups of patients.

Tumor cells frequently express tumor specific antigens. These antigens are potential targets for immunotherapy. Four cancer specific antigens FOXM1, DEPDC1, KIF20A, and URLC10 were identified based on differential expression in GC samples versus normal tissue based on cDNA arrays and immunohistochemical staining. Using computer-based algorithms (BIMAS) for predicting MHC class I/peptide binding, 4 candidate peptide vaccines were constructed. Forkhead box protein M1 (FOXM1) is known to play a key role in cell cycle progression where endogenous FOXM1 expression peaks at S and G2/M phases. Abnormal upregulation of FOXM1 is involved in the oncogenesis of several cancers. Upregulation of FOXM1 promotes oncogenesis through abnormal impact on its multiple roles in cell cycle and chromosomal/genomic maintenance [4]. Ribonucleic acid interference (RNAi) knockdown of DEP domain-containing protein 1 (DEPDC1) expression causes decreased growth in bladder cancer cell lines with an increase in the proportion of cells undergoing apoptosis, as measured by fluorescence activated cell sorter analysis [5]. Kinesin family member 20A (KIF20A) belongs to the kinesin class of microtubule motor proteins, which have critical function in trafficking of molecules and organelles. It has been implicated in pancreatic cancer carcinogenesis and knock down by small interfering RNA (siRNA) led to suppression of tumor growth [6]. Upregulated in lung cancer 10 (URLC10), also known as lymphocyte antigen 6 complex locus K (LY6K), were shown to be over-expressed in several cancers [7]. Tumor suppression was observed in lung cancer and esophageal cancer cell line when URLC10 was knocked down by siRNA.

Angiogenesis is a critical mechanism for tumor progression. Vascular epithelial growth factor (VEGF) is the most potent and specific promoter of angiogenesis known. VEGF activates 2 high-affinity tyrosine kinase receptors, VEFGR1 (flt-1) and VEGFR2 (flk-1/KDR) [8]. The expression of VEGF and VEGFR were strongly correlated with tumor progression and poor prognosis in GC [9]. Vaccines against VEGFR are potentially effective regardless of tumor type. In addition, it could overcome the problems associated with conventional cancer vaccine therapies such as loss of MHC class I molecules and lack of target molecules.

OTSGC-A24 is a HLA-A*24:02-binding peptide vaccine cocktail targeting FOXM1, DEPDC1, KIF20A, URLC10 and VEGFR1. In preclinical models, both down regulation of these targets with siRNA and active vaccination resulted in tumor regression. It has been demonstrated that stimulation of peripheral CD8-positive T cells of healthy individuals (ex-vivo) with these peptides induced cytotoxic T lymphocytes (CTL) with potent cytotoxic activity against target cells having these peptides bound on the cell surface [10]. Furthermore, it has been shown that the CTL clones established from these CTLs specifically recognize HLA-A24-positive cells that endogenously express FOXM1, DEPDC1, KIF20A, or URLC10, respectively in a dose dependent manner [10]. These results suggest that a cancer vaccine therapy with these peptides would induce specific CTLs and exhibit antitumor effect. Vaccine therapy is ideally administered to patients in the adjuvant setting. However, as the long-term effect of OTSGC-A24 in human subjects is not known, we investigated safety, immunogenicity, and optimal scheduling of OTSGC-A24 in patients with GC refractory or intolerable to standard therapy.

## Methods

### Patient population

Patients with histologically confirmed inoperable or metastatic adenocarcinoma of the stomach or lower third of the esophagus refractory to standard therapy, age 21 years or over, and Eastern Cooperative Oncology Group performance status (ECOG PS) 0-2 were eligible. Patients screened positive for HLA-A*24:02 were eligible for study entry. Only subjects with HLA-A*24:02 were eligible for the study because components of OTSCG-A24 were HLA-A*24:02 specific binding peptides. Patients had adequate hematological and organ function and a life expectancy of at least 3 months. Exclusion criteria included major surgery within 28 days prior to enrollment, history of significant gastrointestinal bleeding that required intervention within the month prior to enrollment, previous history of intestinal perforation, symptomatic brain metastasis and uncontrolled intercurrent illnesses. Details and additional inclusion/exclusion criteria are available in the supplementary.

### Ethics, consent and permissions

The study was conducted in accordance with the Declaration of Helsinki, the International Conference on Harmonization guideline on Good Clinical Practice, and applicable local regulatory requirements and laws. All patients provided written informed consent before any study-specific activities were performed. Investigators obtained prospective approval of the study protocol, protocol amendments, informed consent forms, and other relevant documents from the Institutional Review Board: Domain Specific Review Board (DSRB), Reference No: 2010/00575.

### Trial design and treatment

Many cancer vaccine trials have used the traditional “3 + 3 design” and except in very rare situations, a maximum tolerated dose for a cancer vaccine was not identified. The dose-toxicity curves of cancer vaccines are often flat, and the highest dose administered is limited by manufacturing or anatomic issues rather than toxicity. OTSGC-A24 consists of 1 μmol (approximately 1 mg) of OTSGC-A24-Fo, OTSGC-A24-De, OTSGC-A24-Ki, OTSGC-A24-VE1 and OTSGC-A24-Ur (as active pharmaceutical ingredient (API)) and is administered subcutaneously. Apart from OTSGC-A24-Fo, all components of OTSGC-A24 were found to induce specific CTL response at the dose of 1 mg administered at weekly interval [11]. Hence, to evaluate the safety, immunological response and optimal schedule, three cohorts were planned, with 1 mg of OTSGC-A24 being administered at 3-weekly (3w cohort), 2-weekly (2w cohort), and weekly (1w cohort) intervals. A minimum of 3 patients were planned for each cohort, evaluation of CTL induction rate was performed when stage I was completed. If ≥2 of 3 patients attained immune response, the cohort was expanded to 10 patients. This expansion was to allow for better assessment of the immune response rate. The decision to expand the cohort was based on specific CTL induction rate (ELISPOT) by day 28. If ≥2 of 3 patients attained immune response in all 3 dose cohorts, the expansion would only apply to the 2 lower dose cohorts. Recommended phase 2 dose (RP2D) was defined as the lowest dose cohort with ≥2 of 3 patients achieving specific CTL induction.

### Study endpoints and assessments

The study had co-primary efficacy and safety endpoints. The primary efficacy endpoint was antigen specific CTL response rate at 12 weeks. The primary safety endpoint was the overall safety profile, characterized by type, frequency, severity, timing, and relationship to trial treatment of adverse events (AEs) and laboratory abnormalities. Safety was assessed by monitoring AEs during the trial (from initiation of study treatment until at least 28 days after the last dose of OTSGC-A24), by clinical laboratory tests, recording vital signs, 12-lead electrocardiography, and performance status. AEs were graded using National Cancer Institute Common Terminology Criteria for Adverse Events, version 3.0 (NCI CTCAE v 3.0). Dose liming toxicity (DLT) was defined as any of the following occurring during the first 28 days of treatment, when considered related to study treatment: CTCAE grade 4 neutropenia for more than 7 days, CTCAE grade 4 thrombocytopenia, CTCAE grade 3 or higher febrile neutropenia, or any grade 3 or 4 non-hematologic toxicity unless definitive alternative etiology was identified.

Evaluation of antitumor activity was based on objective tumor assessments by investigator review of computed tomography or magnetic resonance imaging scans using Response Evaluation Criteria in Solid Tumors version 1.0. Tumor assessments were performed at baseline and at the end of every 8 weeks (± 1 week) after first dose of OTSGC-A24. Tumor assessment had to be performed at fixed time points regardless of treatment delay. As a consequence of the immunological mechanism of action of OTGSC-A24, considerable time is needed for cancer vaccines to induce immunity after administration, and has been demonstrated that tumors in some subjects treated with cancer vaccines showed early progression followed by subsequent response. Upon evidence of first radiological progression, patients could continue treatment for another 8-week period. Treatment was only discontinued if repeat radiological assessments confirmed clear evidence of progression. Hence, patients could continue with OTSGC-A24 till a confirmatory scan 8 weeks from initial scan showed definitive progression.

### ELISPOT assay

Peripheral blood mononuclear cells (PBMCs) isolated from blood samples from two succeeding collection points (i.e. screening & Day 1 of Pre-Study, Day 29 & 36 of Post 4 W, Day 85 and 92 of Post 12 W and post 12-weekly time-points thereafter) were combined, and treated as one sample to acquire sufficient quantity of PBMCs for analysis. PBMCs were cultured in rIL-2 with each peptide for 14 days and subjected to the ELISPOT assay to detect the antigen-specific T-cell response induced by the vaccination. The positivity of antigen-specific T cell response were classified into negative, 1+, 2+, and 3+ depending on the amount of peptide-specific spots and invariability of peptide-specific spots at different responder/stimulator ratios.

### Assessment of target molecule expression

#### Immunohistochemical (IHC) staining

Ten or more unstained slides of archival paraffin – embedded tumor tissue were obtained to determine the expression of target molecule in either primary or metastatic tumor tissues. Immune-staining was performed by OncoTherapy Science, Inc. to provide information on tumor response and target antigen expression. Antibodies used were rabbit polyclonal antibody FOXM1 (C-20) to FOXM1 (Santa Cruz sc-502), mouse monoclonal antibody 16E9 to DEPDC1 (OncoTherapy Science), rabbit polyclonal antibody KIF20A/RAB6KIFL to KIF20A (Bethyl A300-879A) and mouse monoclonal antibody 3B53G11 to URLC10 (OncoTherapy Science). IHC results were reported for intensity of staining. 3+ was defined as strongly positive, 2+ intermediate, 1+ weakly positive, and 0 as negative.

For patients that achieved positive ELISPOT test, the ex- or in-vivo cell kill activity of active cytotoxic T-lymphocyte in pre-vaccination PBMC and post vaccination PBMC was investigated. PBMCs were expanded ex-vivo to obtain adequate cell number.

### Ex vivo study

In-vitro re-stimulation of peptide-specific CTL was performed for one patient, by obtaining vaccine-induced PBMC and re-simulated with KIF20A based on a protocol previously described [12]. Peptide-specific CTL clone were developed by isolating CD8 T cells and coculturing with EB-3 and Jiyoye cells with IL-2 and anti-CD3 mAb and analysed with ELISPOT assay for specificity followed by CTL clonal expansion. KIF20A-expressing HLA-A24-positive MKN-45 cell line (American Type Culture Collection) was selected as target cell for cytotoxicity assay. Cytotoxicity activity of the specific CTL clone was measured using a calcein-release assay [13]. Resting CD8 T cells from a healthy donor was used as control.

### Statistical design and analyses

Descriptive statistics were used to summarize all patient characteristics, treatment administration/compliance, efficacy endpoints, and safety parameters. Survival was analyzed by the generation of Kaplan Meier curves. Efficacy end points included induction of specific CTL response after vaccination, objective response rate (ORR), progression free survival (PFS), and overall survival (OS). Analyses consisted of Pearson’s chi square tests to test association between categorical variables, and independent sample t-tests and one-way ANOVAs to test association between categorical and numerical variables. Statistical significance was set at two- sided *p* value ≤0.05.

Sample size determination was performed by dosing three subjects at each initial dose. If one or less of three subjects treated at a particular dose showed positive immune response, we could conclude with 90% confidence that the true probability of achieving immune response at the dose unlikely to be greater than 80%. If two or more of three subjects attained immune response, the cohort was expanded to 10 patients. This expansion was to allow for better assessment of the immune response rate. If two or more of three subjects attained immune response in all three dose cohorts, the expansion was only applied to the two lower dose cohorts. This was to keep the immune response rate in the lowest possible dose.

## Results

### Patient characteristics

A total of 24 patients (17 male and 7 female) were enrolled, and all patients received treatment in three cohorts between November 2012 and June 2016. A total of 20 subjects were evaluable for CTL response, 1 subject discontinued study, due to patient preference and 3 subjects had rapid radiological evidence of disease progression before week 4 ELISPOT test. Median age was 56 years (range 34 – 75) and majority (96%) were treated with 2 or more prior lines of therapy. After three patients were treated in each cohort, at 4 weeks, CTL response rates were 33% in 3w cohort, 100% in 2w cohort and 67% in 1w cohort. This lead to expansion of 2w cohort and 1w cohort, with 11 patients eventually being treated in 2w cohort and 10 patients being treated in 1w cohort. Details of patient characteristics are summarized in Table 1.

**Table 1:** Patient characteristics

		N (%)
Sex	Male	17 (71)
	Female	7 (29)
Age	Median	56 years
	Range	34 to 75 years
Histology	Adenocarcinoma	21 (88)
	Others	3 (12)
Stage	III	6 (25)
	IV	18 (75)
Performance Status	0	15 (63)
	1	8 (33)
	2	1 (4)
Number of prior lines of chemotherapy	1	1 (4)
	2 or more	23 (96)

### Safety and tolerability

Safety and tolerability was assessed in 24 patients who were enrolled and received study treatment. No DLT was observed in the population during the DLT evaluation period. One patient died due to progressive disease, one died due to gastric hemorrhage from progressive disease and one due to aspiration pneumonia secondary to gastroesophageal junction stenosis leading from progressive disease.

The most common AEs were decreased appetite (29.2%), myalgia (25.0%), injection site erythema/induration (25.0%), aspartate aminotransferase increase (25.0%), blood alkaline phosphatase increase (25.0%), and diarrhea (21%) which occurred in 5 or more subjects (Table 2). Drug related AEs was experienced in 13 subjects (54.2%): injection site erythema/induration (25.0%) and myalgia (16.7%). In total, 14 subjects (58.3%) experienced AEs with Grade 3 or Grade 4 acute toxicities, with the most common being lung infection (8.3%) and blood alkaline phosphatase increase (8.3%). A majority of the Grade 3 or 4 AEs were not attributed to study drug. Of the two study-drug related Grade 3 AEs, one was anaemia, which was investigated and attributed to bleeding primary progressive gastric tumor, but as one of the drug targets included VEGF, causality to study-drug could not be ruled out. The other grade 3 AE was a transient rise in aspartate transaminase after the first dose of vaccine, which resolved with expectant management. No adverse event with Grade 4 or Grade 5 was observed.

**Table 2:** Adverse Events

	3w Cohort				2w Cohort				1w Cohort				Total			
	(*n* = 3)				(*n* = 11)				(*n* = 10)				(*n* = 24)			
	Any Grade	(%)	Grade 3/4	(%)	Any Grade	(%)	Grade 3/4	(%)	Any Grade	(%)	Grade 3/4	(%)	Any Grade	(%)	Grade 3/4	(%)
Infections																
Lung infection	0	(0)			2	(18)	2	(18)	0	(0)			2	(8)	2	(8)
URTI	1	(33)			1	(9)			0	(0)			2	(8)		
Other infection	0	(0)			0	(0)			3	(30)	1	(10)	3	(13)	1	(4)
Gastrointestinal																
Diarrhoea	0	(0)			3	(27)			2	(20)			5	(21)		
Dyspepsia	0	(0)			3	(27)			1	(10)			4	(17)		
Constipation	1	(33)			0	(0)			2	(20)			3	(13)		
Abdominal pain	2	(66)			0	(0)			1	(10)	1	(10)	3	(13)	1	(4)
Nausea	1	(33)			1	(9)			1	(10)			3	(13)		
Vomiting	2	(66)			0	(0)			1	(10)			3	(13)		
Dysphagia	0	(0)			0	(0)			2	(20)			2	(8)		
Reflux	2	(66)			0	(0)			0	(0)			2	(8)		
Haematological																
Anaemia	1	(33)	1	(33)	0	(0)			1	(10)			2	(8)	1	(4)
Hepatic																
ALT increase	1	(33)			1	(9)			2	(20)			4	(17)		
AST increase	1	(33)			2	(18)			3	(30)	1	(10)	6	(25)	1	(4)
GGT increase	0	(0)			0	(0)			1	(10)	1	(10)	1	(4)	1	(4)
ALP increase	1	(33)			2	(18)	1	(9)	3	(30)	1	(10)	6	(25)	2	(8)
Transaminitis	0	(0)			0	(0)			1	(10)	1	(10)	1	(4)	1	(4)
Hyperbilirubinemia	0	(0)			0	(0)			2	(20)			2	(8)		
Injection reaction																
Site erythema/ induration	1	(33)			3	(27)			2	(20)			6	(25)		
Site ulcer	0	(0)			0	(0)			1	(10)			1	(4)		
Respiratory																
Cough	1	(33)			0	(0)			2	(20)			3	(13)		
Dyspnea	0	(0)			0	(0)			1	(10)	1	(10)	1	(4)		
Neoplastic																
Tumor haemorrhage	0	(0)			0	(0)			1	(10)	1	(10)	1	(4)	1	(4)
Cancer pain	0	(0)			3	(27)	1	(9)	1	(10)			4	(17)	1	(4)
Others																
Malaise	0	(0)			1	(9)			1	(10)			2	(8)		
Peripheral edema	0	(0)			0	(0)			2	(20)			2	(8)		
Pyrexia	1	(33)			1	(9)			0	(0)			2	(8)		
Dizziness	0	(0)			0	(0)			1	(10)			1	(4)		
Loss of appetite	1	(33)			3	(27)			3	(30)			7	(29)		
Hypokalemia	1	(33)			1	(9)			1	(10)			3	(13)		
Hyponatremia	0	(0)			2	(18)	1	(9)	0	(0)			2	(8)	1	(4)
Hyperkalemia	0	(0)			2	(18)	1	(9)	0	(0)			2	(8)	1	(4)
Pruritus	0	(0)			0	(0)			2	(20)			2	(8)		
Myalgia	0	(0)			3	(27)			3	(30)			6	(25)		
Urteric Stenosis	0	(0)			0	(0)			1	(10)	1	(10)	1	(4)	1	(4)
Fatigue	0	(0)			2	(18)			0	(0)			2	(8)		

### Efficacy

Analysis of efficacy was conducted on a total of 20 subjects (3w cohort: 3 subjects, 2w cohort: 8 subjects and 1w cohort: 9 subjects) out of 24 treated subjects.

#### CTL responses

In the first phase of the study, with 3 patients treated in each cohort, CTL response was measured at 28 days after first dose of OTSGC-A24. Response rates were 33% in 3w cohort, 100% in 2w cohort and 67% in 1w cohort. This led to cohort expansion of 2w cohort and 1w cohort. Positive CTL responses (3+) after vaccination were observed in 15 patients (75%) at 4 weeks: 3w cohort (33%), 2w cohort (88%), 1w cohort (78%). At 12 weeks, 18 patients had responded (90%); 3w cohort (100%), 2w cohort (100%), 1w cohort (78%). By antigens, in FOXM1, positive responses after vaccination at 12 weeks were the highest (Total: 18 subjects [90.0%], 3w cohort: 3 subjects [100.0%], 2w cohort: 8 subjects [100.0%], 1w cohort: 7 subjects [77.8%]). The second highest response was to URLC10 (Total: 13 subjects [65.0%], 3w cohort: 1 subject [33.3%], 2w cohort: 5 subjects [62.5%] and 1w cohort: 7 subjects [77.8%]) (Table 3).

**Table 3:** CTL Positive Response

	3w Cohort			2w Cohort			1w Cohort			Total cohort		
	*N* = 3 (%)			*N* = 8 (%)			*N* = 9 (%)			*N* = 20 (%)		
Week	0	4	12	0	4	12	0	4	12	0	4	12
FOXM1	0 (0)	1 (33)	3 (100)	0 (0)	7 (88)	8 (100)	0 (0)	7 (78)	7 (78)	0 (0)	15 (75)	18 (90)
DEPDC1	0 (0)	0 (0)	0 (0)	1 (13)	2 (25)	4 (50)	0 (0)	2 (22)	3 (33)	1 (5)	4 (20)	7 (35)
KIF20A	0 (0)	0 (0)	1 (33)	0 (0)	3 (38)	4 (50)	0 (0)	3 (33)	3 (33)	0 (0)	6 (30)	8 (40)
URLC10	0 (0)	1 (33)	1 (33)	0 (0)	4 (50)	5 (63)	1 (11)	7 (78)	7 (78)	1 (5)	12 (60)	13 (65)
VEGFR1	0 (0)	0 (0)	1 (33)	0 (0)	2 (25)	2 (25)	0 (0)	0 (0)	0 (0)	0 (0)	2 (10)	3 (15)
Any of above	0 (0)	1 (33)	3 (100)	1 (13)	7 (88)	8 (100)	1 (11)	7 (78)	7 (78)	2(10)	15 (75)	18 (90)
*p* value (vs. 2w)	0.35	0.03	0.41	NA	NA	NA	1	0.88	0.51	NA	NA	NA

#### Response rate and survival

**Fig. 1:**
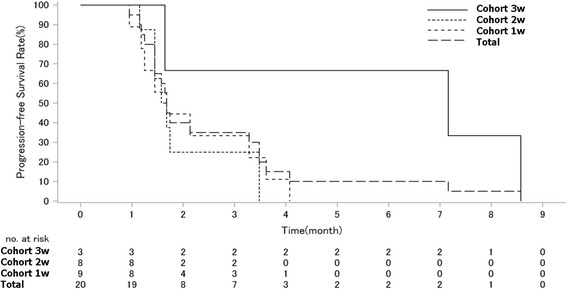
Kaplan-Meier Curve of PFS

**Fig. 2:**
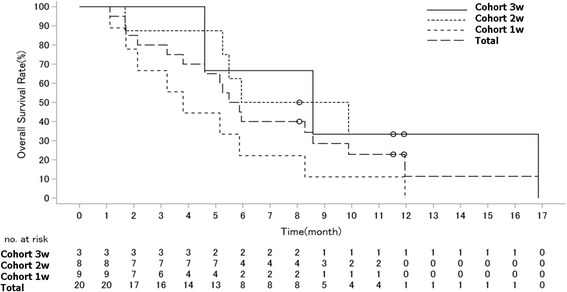
Kaplan-Meier Curve of OS

No complete response (CR) or partial response (PR) was seen in the cohort. Stable disease (SD) was the best response in 8 patients (40%) (3w cohort: 2 (67%), 2w cohort: 2(25%), 1w cohort 4(44%)). The median PFS for the cohort was 1.7 months (95% CI: 1.4 to 3.5). By cohorts, median PFS was 3w cohort 7.2 months (95% CI 1.6 to 8.6), 2w cohort 1.6 months (95% CI 1.4 to 3.5) and 1.7 months (95% CI 1.2 to 3.3) (Fig. 1). The median OS for the cohort was 5.7 months (95% CI 3.8 to 8.6). By cohorts, median OS was 3w cohort 8.6 months (95% CI 4.6 and 16.9), 2w cohort 7.9 months (95% CI 5.3 to NR), and 1w cohort 3.8 months (95% CI 2.1 to 5.9) (Fig. 2**)**.

#### Correlation between CTL response and survival

Those with a CTL response of (−) had a median overall survival of 1.1 months. Those with a CTL response of (1+) was 5.2 months and (3+) was 5.9 months. Although numerically longer OS was observed more in (1+), (2+) and (3+) compared with that of (−), correlation between specific CTL response and OS was not clearly observed (*r* = 0.356). There was no correlation between individual antigen specific CTL response and OS.

#### Immunohistochemistry

IHC was performed for FOXM1, DEPDC1, KIF20A and URLC10 in 11 samples. For FOXM1, 73% (8 of 11) of samples stained 2+ and 27% stained 1+ while none were negative. DEPDC1 had 55% (6 of 11) that stained 1+ while the rest were negative. KIF20A had 18% that stained negative, 64% (7 of 11) stained 1+, and 18% stained 2+. For FOXM1, DEPDC1 and KIF20A there were no 3+ results. IHC for URLC10 revealed the following results 3+:36%, 2+: 45% (5 of 11), 1+: 9% and 0: 9%.

#### Correlation between IHC and CTL response

For FOXM1, all cases stained positive (1+ or more) and developed a CTL response by 12 weeks. There is poor correlation between IHC and CTL response for DEPDC1 and KIF20A. For DEPDC1, and KIF20A, one of three and one of two cases which were IHC negative, respectively, mounted a positive CTL response by day 28. Only for URLC10, there was strong correlation between IHC staining and CTL response. Cases that had an IHC score of 0 or 1 did not mount a response. All cases that had an IHC score of 3+ mounted a response, while only one (of five) cases with an IHC score of 2+ did not mount a response.

#### Ex vivo study

**Fig. 3:**
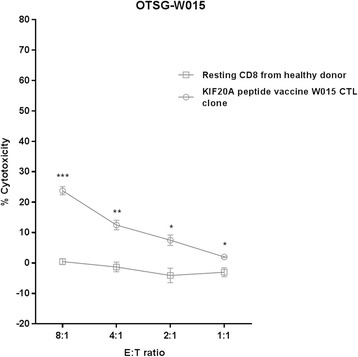
Ex vivo study. Cytotoxicity activity of KIF20A peptide-specific CTL clone against MKN-45 cell lines (open circle) or resting CD8 cells (open square). KIF20A peptide-specific CTL clone exert significantly higher cytotoxicity activity than the resting CD8 T cells. E/T ratio, effector cell (CTL clone or resting CD8)/target cell (tumor cell) ratio. *** *p* < 0.001, ** *p* < 0.01, * *p* < 0.05

Cytotoxicity activity of KIF20A peptide-specific CTL clone against MKN-45 cell lines or resting CD8 cells is depicted in Fig. 3. KIF20A peptide-specific CTL clones exerted significantly higher cytotoxicity activity than the resting CD8 T cells.

## Discussion

Immune evasion is now recognized to play a key role in the organization and development of cancer, and has been included as a hallmark of cancer [14]. The cancer-immunity cycle describes several steps starting from release of cancer cell antigens from cell death, cancer antigen presentation by dendritic cells and other antigen-presenting cells (APCs), priming and activation by APCs and T cells, trafficking of T cells to tumors (CTLs), infiltration of T cells into tumors (CTLs and endothelial cells), recognition of cancer cells by T cells and eventually killing of cancer cells [15]. Increased immune cell infiltration has been associated with improved prognosis in various cancer types [16]. The immune contexture in gastric cancer predicts prognosis; the type and density of tumor infiltrating T lymphocytes (TILs) are an independent predictor of lymph node metastasis and overall survival after gastrectomy [17]. Moreover, the immune contexture of various populations is different, with Asians having lower CD68/CD3 ratios and higher overall survival [18]. Several integrated platforms for cancer immune profiling are now emerging to select patients better various immunotherapy [19]. Becht et al. describes three immune subgroups – a) immunogenic: with good prognosis and characterized by abundant TILs, mature dendritic cells, CTLs, M1 macrophages and IFN- ƴ and CXCL13; b) immune neglected: with intermediate prognosis, characterized by lack of TILs, CTLs and cytokines and downregulation of MHC Class I; c) Inflammatory: poor prognosis, characterized by lack of tertiary lymphoid structures, abundant lymphocytes, immature dendritic cells, M2 macrophages, highly vascularized and PD-L1 expression [20]. It is now hypothesized that while the immunogenic and inflammatory sub-groups will benefit from immune checkpoint blockade, the immune-neglected sub-group may benefit from cancer vaccine therapy.

The primary objective of this study was to evaluate the safety, immunogenicity, and optimal scheduling of OTSGC-A24 in patients with GC refractory or intolerable to standard therapy. Three cohorts were investigated, with 1 mg of OTSGC-A24 being administered at 3-weekly (3w cohort), 2-weekly (2w cohort), and weekly (1w cohort) intervals. No DLTs were observed, and the most common drug related toxicities were injection site related erythema or induration which was mild and easily reversible. Recommended phase 2 dose (RP2D) was defined as the lowest dose cohort with patients achieving specific CTL induction, and this was established as 1 mg OTSGC-A24 sub-cutaneous, every 2 weeks. No responses were seen, and 40% had best response of stable disease. Median progression free survival was 1.7 months and median overall survival was 5.7 months. Immunohistochemistry analysis of the various peptides revealed that URLC10 IHC appeared to correlate with CTL response to URLC10, and may potentially be used as a predictive biomarker for patient selection in future trials involving URLC10 vaccines.

The first cancer vaccine to be approved was sipuleucel-T, in 2010 by the Food and Drug Administration (FDA) [21]. This was followed by FDA approval of oncolytic virus therapy, talimogene laherparepvec for the treatment of metastatic melanoma in 2015 [22]. There has been a drive to investigate the role of cancer vaccines in various tumor groups, including gastrointestinal cancers. A cancer vaccine trial involving URLC10 and VEGFR1 has been investigated in advanced gastric cancer using a fixed dose model [23]. No responses were noted, and 30% had stable disease. HLA genotype was investigated after enrollment, and HLA-A*2402 positive and negative patients had similar median survival of 4.2 and 3.6 months (*p* = 0.92). Overall median survival of the cohort was 3.9 months. LY6K peptide vaccine was studied in a fixed dose in six patients with advanced gastric cancer [24]. No responses were seen and stable disease in 50%. HLA-A*2402-positive patients with advanced gastric cancer were treated with cisplatin, S-1 and a vaccine consisting of VEGFR1-1084 and VEGFR-169 [25]. Twenty two patients were treated and 55% demonstrated a partial response. Median time to progression was 9.6 months and overall survival was 14.2 months. A five-epitope peptide vaccine of MPHOSPH1, TTK, KOC1, URLC10 and DEPDC1 was tested in combination with cyclophosphamide [26], with promising early results with respect to CTL response and tolerability. As several cancer vaccine trials previously designed in a dose-escalation manner did not achieve MTD [27, 28], most contemporary studies utilize a fixed dose regimen [23–25, 29]. Our trial capitalized on this advantage of cancer vaccines, and focused instead on establishing an RP2D based on CTL response, rather than conventional MTD. Similar to other gastric cancer vaccine studies, drug related toxicities were minimal and but single-agent tumor response could not be demonstrated. Median PFS and OS was also comparable to other studies. Fujiwara et al., report a study of gastric cancer patients treated with a vaccine with DEPDC1, URLC10, FOXM1, KIF20A and VEGFR1 in a weekly regimen [30]. This trial did not select for HLA-A*2402 specific patients, but analysed patients based on HLA status. Positive CTL responses specific for URLC10, DEPDC1, KIF20A, FOXM1 and VEGFR1 were observed in 90, 60, 60, 100 and 55% respectively at 8 weeks post vaccination, which is relatively similar to our studies results at 12 weeks. There was no difference in survival between HLA-A*2402 positive and negative patients, and no objective responses seen. Key differences between the two studies include a) our study tests varying schedules of vaccination to assess the optimal regimen, which has not been identified previously, and was the primary objective of this study b) our study included only HLA-A*2402 patients, making it a more homogenous population to assess clinical activity.

Cancer vaccine development now lags behind immune-checkpoint inhibitor therapies in gastric cancer, with several studies now maturing with promising results. Nivolumab was compared against placebo in advanced gastric cancer patients who had received at least two prior regimens and demonstrated an improved in overall survival from 4.1 to 5.3 months, with an objective response rate of 11% [31]. Pembrolizumab demonstrated a response of 22%, with a median overall survival of 11.4 months [32]. Trials are currently ongoing investigating immune-checkpoint inhibitors in combination with chemotherapy and other agents. It is likely that future of cancer vaccine therapy will need to incorporate immune checkpoint inhibitors, either as combination or sequential therapy. Simultaneously, several groups are working on novel formulations of the adjuvant to improve clinical efficacy [33].

## Conclusion

OTSGC-A24 peptide vaccine is safe and well tolerated, and demonstrates significant CTL responses. Further studies are required to identify the sub-group of patients who will benefit from this therapy, and its role in gastric cancer management.

## Abbreviations

AE: Adverse events
APC: Antigen presenting cell
CR: Complete response
CTL: Cytotoxic T lymphocyte
DEPDC1: DEP domain-containing protein 1
DLT: Dose limiting toxicity
ECOG PS: Eastern Cooperative Oncology Group performance status
FDA: Food and Drug Administration
FOXM1: Forkhead box protein M1
GC: Gastric cancer
HLA: Human leukocyte antigen
IFN: Interferon
IHC: Immunohistochemical staining
KIF20A: Kinesin family member 20A
LY6K: Lymphocyte antigen 6 complex locus K
MHC: Major histocompatibility complex
MTD: Maximum tolerable dose
NCI CTCAE v 3.0: National Cancer Institute Common Terminology Criteria for Adverse Events, version 3.0
ORR: Objective response rate
OS: Overall survival
PFS: Progression free survival
PMBC: Peripheral blood mononuclear cells
PR: Partial Response
RECIST: Response Evaluation Criteria in Solid Tumors
RP2D: Recommended phase 2 dose
SD: Stable disease
TIL: Tumor infiltrating lymphocyte
URLC10: Upregulated in lung cancer 10
VEGF: Vascular epithelial growth factor
VEGFR: Vascular epithelial growth factor receptor
